# Macrophage-stimulated microRNA expression in mural cells promotes transplantation-induced neointima formation

**DOI:** 10.18632/oncotarget.16279

**Published:** 2017-03-16

**Authors:** Xiaotong Guo, Mengyao Sun, Chaochao Dai, Xun Zhang, Qihui Yin, Jiaqi Ling, Xinyue Li, Xiao Wu, Fan Jiang, Jianli Wang

**Affiliations:** ^1^ Department of Pathology and Pathophysiology, School of Basic Medicine, Shandong University, Jinan, Shandong Province, China; ^2^ Key Laboratory of Cardiovascular Remodeling and Function Research, Chinese Ministry of Education and Chinese Ministry of Health, Qilu Hospital of Shandong University, Jinan, Shandong Province, China; ^3^ State-Shandong Province Joint Key Laboratory of Translational Cardiovascular Medicine, Qilu Hospital of Shandong University, Jinan, Shandong Province, China

**Keywords:** transplant vasculopathy, neointima, macrophage, microRNA, mural cells

## Abstract

In this study, we tested the possibility that macrophages might contribute to neointima formation by stimulating microRNA expressions in mural cells. Thoracic aortas from F344 rats were transplanted into recipient Lewis rats. Clodronate liposome was used for *in vivo* macrophage depletion. Using miR-21 as a prototypic example of vascular enriched microRNA, we showed that macrophage depletion reduced the expression level of miR-21, which was upregulated in the allograft. This effect of macrophage depletion was accompanied by attenuations in neointimal hyperplasia and transplantation-induced vascular inflammation. Using *in vitro* assays, we identified that macrophages might stimulate miR-21 expression in smooth muscle cells and adventitial fibroblasts via the release of tumor necrosis factor-α. We also showed that silencing of miR-21 suppressed tumor necrosis factor-induced proliferation, migration, and inflammatory responses in mural cells. Our results suggest that macrophage may promote transplantation-induced neointima formation by stimulating miR-21 expression in vascular mural cells, which promotes mural cell proliferation, migration and/or inflammation. Moreover, we have established that tumor necrosis factor-α has a major role in mediating this paracrine process.

## INTRODUCTION

Acute rejection of allografts following solid organ transplantation can be prevented pharmacologically by various immunosuppressive agents [1]. In contrast, chronic rejection is still a clinical challenge that limits the long-term survival of transplanted organs [2]. A major pathogenic mechanism underlying chronic rejection is the development of arterial transplant vasculopathy [2–5]. For example, population studies have suggested that around 90% of patients receiving heart transplantation will develop transplant vasculopathy within 10 years [6]. Transplant vasculopathy is also known as transplant arteriosclerosis, which is characterized by progressing neointimal hyperplasia, luminal stenosis, and finally ischemic graft failure [3–5].

It has been recognized that proliferation and migration of vascular smooth muscle cells (VSMCs) driven by multiple local and systemic factors have a pivotal role in transplant vasculopathy [3–5, 7]. Although these processes are also involved in the pathogenesis of angioplasty-induced restenosis, there are fundamental differences between these two diseases. Unlike angioplasty-induced lesions, transplant vasculopathy occurs in a diffuse manner and may cover the entire length of the arterial tree [5, 6]. Hence, interventional techniques are unsuitable for treating transplant vasculopathy. More importantly, it is clear that the initiating factor for angioplasty-induced restenosis is the massive mechanical denudation of luminal endothelium; however, this is not the primary etiology of transplant vasculopathy. The biological mechanisms of transplant vasculopathy are not completely understood; as a consequence, an effective and safe treatment strategy targeting this process is still lacking [8].

Using a rat model of aortic allograft transplantation [9–13], we have discovered that activation of the adventitial layer is the initial pathological event which occurs prior to the appearance of neointima [14], suggesting that the adventitia may have important roles in the pathogenesis of neointimal hyperplasia in response to transplantation. This early adventitial activation is characterized by cellularization and thickening of the layer, increased proliferation and trans-differentiation of fibroblasts, expression of multiple pro-inflammatory molecules, and increased expression of NADPH oxidase [14, 15]. Interestingly, we have identified that CD68^+^ macrophages, but not CD3^+^ T lymphocytes, are the predominant type of leukocytes infiltrating the activated adventitia [14].

To further clarify the functional importance of macrophage infiltration in transplantation-induced arterial remodeling and the underlying mechanisms, in the present study, we examined the effects of macrophage depletion using clodronate liposome. It is noted that mounting evidence has pointed to a critical role of microRNAs (miRNAs) in the pathogenesis of arterial remodeling [16–24]. These miRNAs may exert either protective or detrimental effects mainly by modulating the proliferation, migration and/or phenotype differentiation of vascular smooth muscle cells. Macrophages secrete high levels of various inflammatory cytokines, while the expressions of many vascular tissue-enriched miRNAs are regulated by the pro-inflammatory cue [25, 26]. Based on these data, therefore, we propose that the infiltrating macrophage may contribute to transplantation-induced neointima formation by stimulating miRNA expressions in vascular mural cells. In particular, we selected miR-21 as a target to prove this concept, because miR-21 has been shown to have crucial effects in the pathogenesis of neointima formation in angioplastic and vein graft models [19, 27–30].

## RESULTS

### Macrophage depletion inhibits neointimal growth and vascular inflammation

**Figure 1:**
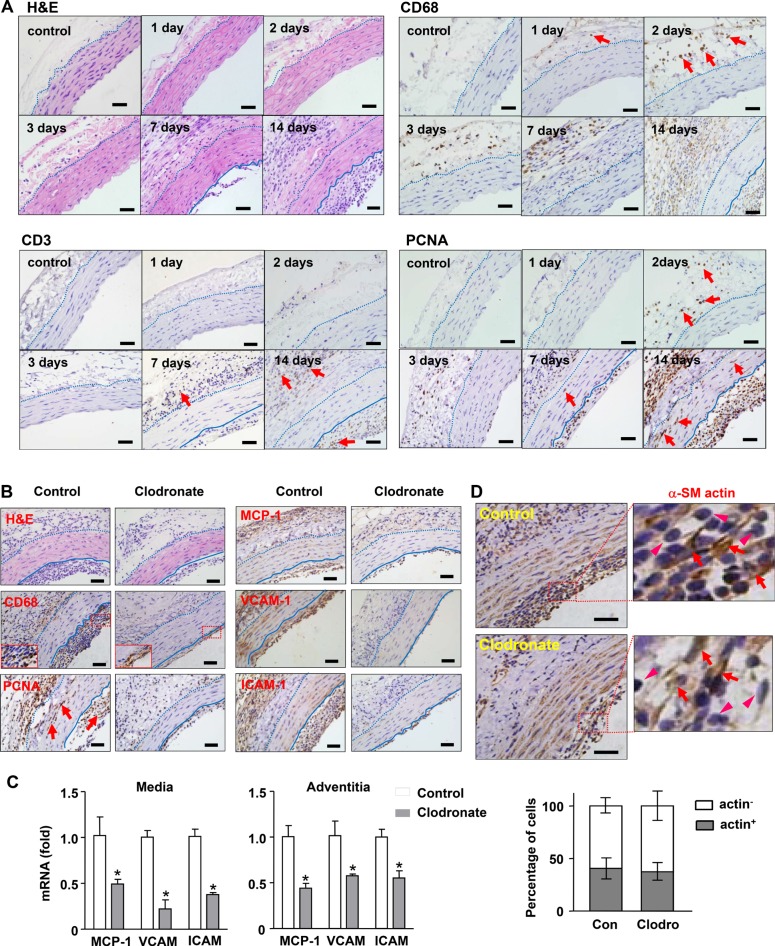
Effects of macrophage depletion with clodronate liposomes on transplantation-induced arterial remodeling and inflammation (**A**) Time course of early adventitial macrophage infiltration and subsequent neointimal hyperplasia revealed by hematoxylin and eosin (H&E) staining and immunohistochemistry. Macrophages were stained with anti-CD68; T cells stained with anti-CD3; and proliferating cells stained with anti-PCNA. (**B**) Histology images showing that macrophage depletion inhibited the growth of neointima, diminished infiltrating macrophages in the adventitia, reduced the number of PCNA^+^ cells, and reduced inflammatory responses (samples of day 14). (**C**) Effects of clodronate on the expression levels of various inflammatory molecules measured with qPCR. (**D**) Proportion of smooth muscle cells (stained with anti-a-SM actin, indicated by arrows) and non-muscle cells (arrowheads) in the neointima of untreated and clodronate-treated vessels. Data are mean ± SD. *P < 0.05 versus control, unpaired *t-test*, *n* = 6. Dashed lines indicate the border of adventitia and media; the solid lines indicate the border between media and neointima. Bar represents 50 μm.

In our previous study, we found that adventitial macrophage infiltration was present as early as 3 days after aorta transplantation. Here we extended the observation to earlier time points. We demonstrated that individual macrophages (CD68^+^) could be detected at 24 hrs, and cell clusters were obvious at 48 hrs (Figure 1A). The population of T cells (CD3^+^) remained minor up to 7 days (Figure 1A). Staining of the cell proliferation marker PCNA showed that proliferating cells also appeared first in the adventitia (Figure 1A). At later stages, PCNA^+^ cells were abundantly present in the adventitia and neointima; the media also contained numerous PCNA^+^ cells. Macrophages were totally absent in the tunica media at any time points examined (Figure 1A). Under the present experimental settings, neointima was barely detectable at day 7, which became large and consistent at day 14 (Figure 1A). The neointima also contained numerous PCNA^+^ cells (Figure 1A).

To define the functional importance of macrophage infiltration in transplantation-induced neointima formation, we depleted peripheral macrophages with clodronate liposomes. We demonstrated that macrophage depletion significantly inhibited the growth of neointima, without any effect on the tunica media (Figure 1B and Table 1). We confirmed that clodronate reduced the abundance of infiltrating macrophages in the adventitia (41 ± 10% in control versus 17 ± 12% in treated specimens, *P* < 0.05, *n* = 4) (Figure 1B). However, the clodronate effect on macrophages in the neointima was insignificant (59 ± 4% versus 56 ± 5%, *P* > 0.05), although the total size of neointima was decreased. This phenomenon suggested that adventitial macrophages might mainly originate from the peritoneal cavity, because intra-peritoneal administration of clodronate depleted peritoneal macrophages more efficiently than circulating monocytes/macrophages. It should be noted that the effect of clodronate on macrophage depletion could be through both peritoneal exposure and its systemic actions. Clodronate treatment also reduced the number of PCNA^+^ cells in all three layers of the vessel wall (Figure 1B and Supplementary Figure I). Moreover, macrophage depletion significantly inhibited vascular inflammatory response as evidenced by the reduced immunoreactivities of MCP-1, VCAM-1 and ICAM-1 in the vessel wall (Figure 1B). We further confirmed the effects of clodronate on vascular inflammation with qPCR. We demonstrated that in both of tunica media and adventitia, the expression levels of MCP-1, VCAM-1 and ICAM-1 were all significantly reduced in clodronate-treated grafts (Figure 1C).

**Table 1:** Effects of macrophage depletion by clodronate on transplantation-induced arterial remodeling

	Intima (μm)	Media (μm)	I/M ratio
Control	54.7 ± 3.7	103.4 ± 5.5	0.53 ± 0.05
Clodronate	22.3 ± 2.9*	100.5 ± 3.7	0.22 ± 0.03*

Data of intimal and medial thickness, and intima/media ratio were presented. Data are mean ± SD. *P < 0.05 versus control, unpaired *t*-test, *n =* 6.

The neointimal layer contained both macrophages (~ 50% CD68^+^ cells) and SMCs (~ 40% SM-actin^+^ cells). Since the last dose of clodronate was given on day 7, it was possible that the observed reduction of neointima at day 14 was due to depletion of macrophages from the blood. To address this question, we stained vessel sections with the smooth muscle cell marker α-SM actin. If the reduced neointima was predominantly caused by removal of macrophages, then the relative proportion of SMCs would be increased. However, our results revealed that the relative proportions of actin^+^ and actin^−^ cells remained similar in treated and untreated vessels (39.2% versus 37.3% for actin^+^ cells, *P* > 0.05) (Figure 1D).

### Macrophage depletion suppresses miR-21 expression in transplanted vessels

**Figure 2:**
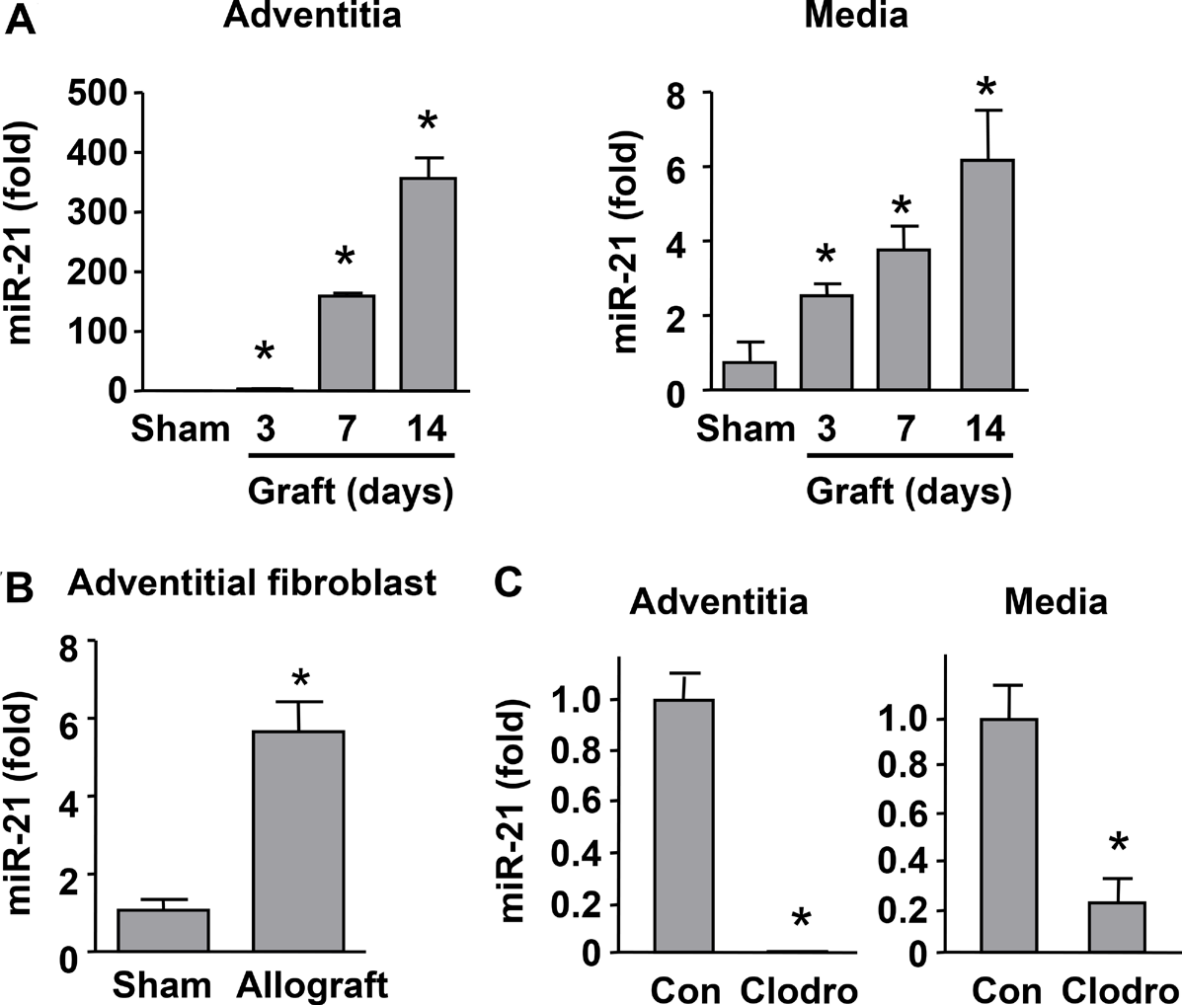
Macrophage depletion suppressed miR-21 expression in transplanted vessels (**A**) Time-dependent increases in miR-21 expression in the media and adventitia of allografts as compared to sham measured with qPCR. (**B**) Expression levels of miR-21 in adventitial fibroblasts isolated from sham and allograft vessels at day 14. (**C**) Macrophage depletion with clodronate significantly reduced the expression levels of miR-21 in the medial and adventitial layers of transplanted vessels at day 14. Data are mean ± SD. *P < 0.05 versus sham or control (Con), unpaired *t-test* or one-way ANOVA as appropriate, *n* = 4.

To clarify whether the expression of miR-21 was associated with transplantation-induced arterial remodeling, and whether the level of miR-21 was affected by macrophage depletion, we measured miR-21 expression using qPCR. Since miR-21 was reported to be expressed by many types of cells including fibroblasts, SMCs and macrophages, we thus tested the tunica media and adventitia independently. We showed that there were time-dependent increases in miR-21 expression in both media and adventitia from day 3 (Figure 2A). Moreover, we isolated primary fibroblasts from sham and allograft adventitial tissues, and showed that miR-21 expression was significantly upregulated in graft-derived fibroblasts (Figure 2B). Then we demonstrated that macrophage depletion with clodronate significantly reduced the expression levels of miR-21 in the medial and adventitial layers of transplanted vessels (Figure 2C). In the present rat model, we confirmed that macrophages were not present in the medial layer throughout the experimental period. Hence our data suggest that macrophage depletion can down-regulate miR-21 expression in medial SMCs. Although we could not clearly distinguish miR-21 expression in adventitial fibroblasts and macrophages, the data in Figure 2B support that fibroblasts contribute to, at least partially, the increased adventitial miR-21 expression in transplanted vessels.

### MiR-21 antagomir ameliorates neointimal hyperplasia *in vivo*

**Figure 3:**
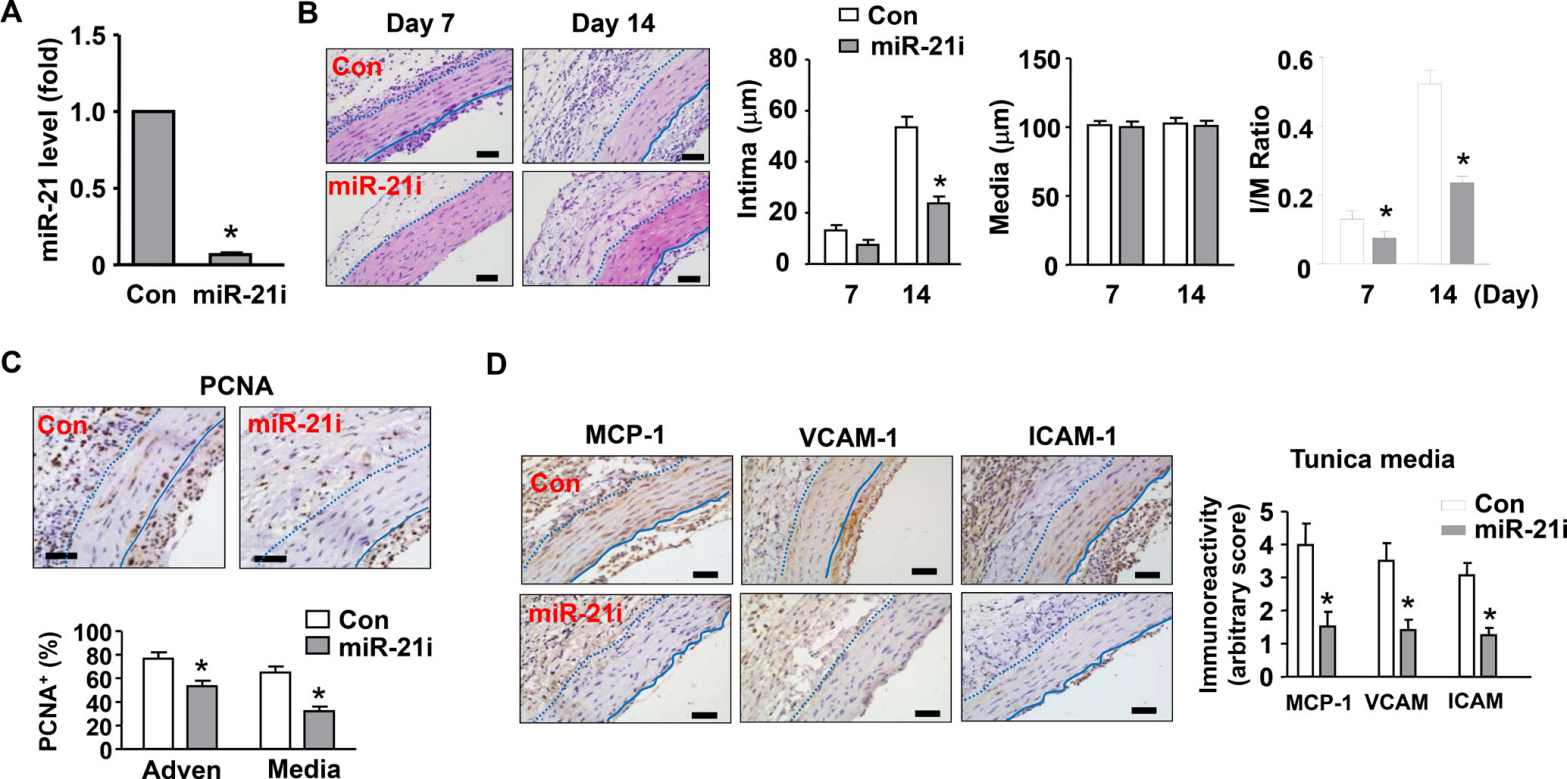
Effects of a miR-21 antagomir (miR-21i) on neointima formation *in vivo* (**A**) Silencing efficiency of the miR-21 antagomir validated in cultured fibroblasts using qPCR. (**B**) Histology images and corresponding quantitative data showing that miR-21 antagomir inhibited neointima formation as compared to vessels treated with a non-targeting control sequence. (**C**) Effects of miR-21 antagomir on the abundance of proliferating (PCNA^+^) cells in the tunica media and adventitia. (**D**) Effects of miR-21 antagomir on vascular inflammation in the allograft as revealed by immuno-staining of MCP-1, VCAM-1, and ICAM-1. The bar graph shows semi-quantitative data of the immunoreactivity in the tunica media. An arbitrary score of 1 (weakest expression) to 5 (strongest expression) was assigned to each section in a blind manner. Data are mean ± SD. *P < 0.05 versus control (Con), unpaired *t-test* or one-way ANOVA as appropriate, *n* = 6.

To determine whether miR-21 had a role in transplantation-induced arterial neointimal hyperplasia, we treated the vessels with local application of an antagomir of miR-21 as described [31]. The silencing efficiency of the miR-21 antagomir was validated with qPCR in cultured fibroblasts (Figure 3A). As compared to vessels treated with a non-targeting control sequence, vessels treated with miR-21 antagomir exhibited a significant reduction in the thickness of neointima (Figure 3B). Moreover, we observed that miR-21 inhibition also reduced the abundance of proliferating (PCNA^+^) cells in both of the tunica media and adventitia (Figure 3C). Immuno-staining for MCP-1, VCAM-1, and ICAM-1 demonstrated that miR-21 antagomir significantly reduced the inflammatory response in tunica media and adventitia of the allograft (Figure 3D). We also performed fluorescent staining of endothelial cells using DyLight 594-labeled *Lycopersicon* lectin (from Vector Laboratories, Burlingame, CA, USA) in aortic cross-sections, and found that clodronate liposome or miR-21 antagomir had no obvious effect on the integrity of the endothelial layer (Supplementary Figure II).

### Macrophage stimulates miR-21 expression in vascular mural cells via a paracrine mechanism

**Figure 4:**
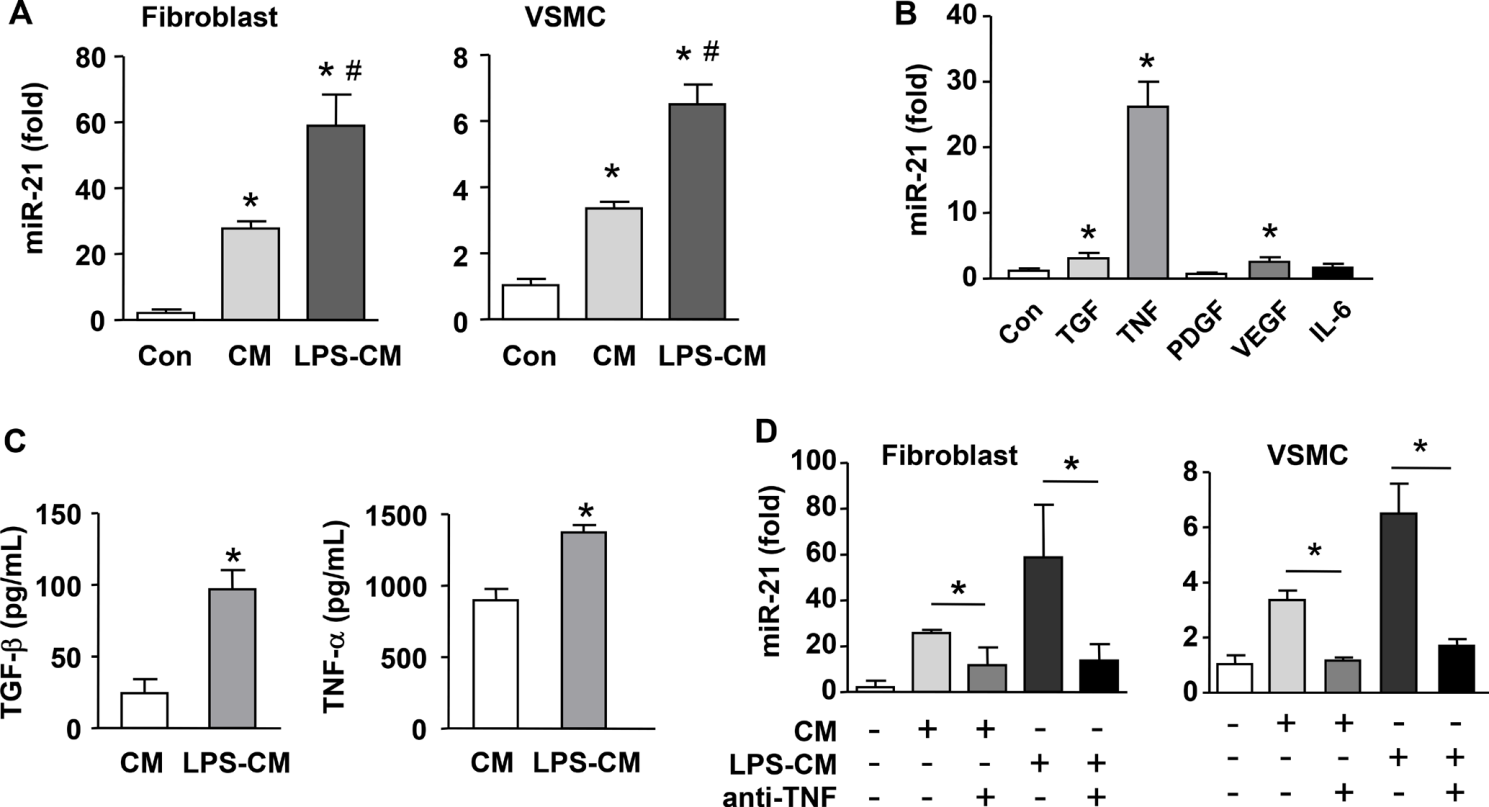
Macrophage stimulates miR-21 expression in cultured vascular mural cells via a paracrine mechanism (**A**) Conditioned medium (CM) from both unstimulated and LPS-pretreated macrophages (LPS-CM) stimulated miR-21 expression in vascular smooth muscle cells (VSMCs) and primary adventitial fibroblasts. (**B**) Screening with qPCR for potential macrophage-derived cytokines that could modulate miR-21 expression tested in NIH3T3 cells. (**C**) Quantification of the concentration of TNF-α and TGF-b in CM and LPS-CM using ELISA. (**D**) The stimulating effects of conditioned medium on miR-21 expression were blocked by a neutralizing antibody to TNF-α in primary fibroblasts and VSMCs. Data are mean ± SD. *P < 0.05 versus Con, ^#^*P* < 0.05 versus CM, one-way ANOVA, *n* = 3.

To clarify whether macrophages could stimulate miR-21 expression in vascular mural cells, we first tested the effects of macrophage-conditioned medium on miR-21 expression in primary adventitial fibroblasts and VSMCs. We demonstrated that the conditioned medium from unstimulated macrophages and LPS-activated macrophages significantly upregulated miR-21 expression in both cells (Figure 4A). To identify the factor(s) that was responsible for this paracrine regulatory effect, we screened the effects of several potential macrophage-derived cytokines on miR-21 expression in NIH3T3 cells, including TGF-β, TNF-α, VEGF, PDGF and IL-6. We found that TNF-α drastically increased miR-21 expression (Figure 4B). Although TGF-β and VEGF also significantly elevated the level of miR-21, their effects were much smaller than that of TNF-α. In contrast, PDGF or IL-6 showed no effects. We also confirmed that TNF-α treatment upregulated miR-21 expressions in primary fibroblasts and VSMCs (Supplementary Figure III). We directly measured the concentration of TNF-α in the conditioned medium, and verified that TNF-α was constitutively produced by macrophages and was increased after LPS stimulation; the level of TNF-α in the conditioned medium was ~10-fold higher than that of TGF-β (Figure 4C). To further corroborate the paracrine effect of macrophage on mural cell miR-21 expression, we treated the conditioned medium with a neutralizing antibody to TNF-α; we demonstrated that the stimulating effects of conditioned medium on miR-21 expression were blocked by the neutralizing antibody in both fibroblasts and VSMCs (Figure 4D).

### Role of miR-21 in inflammatory responses in VSMCs and fibroblasts

**Figure 5:**
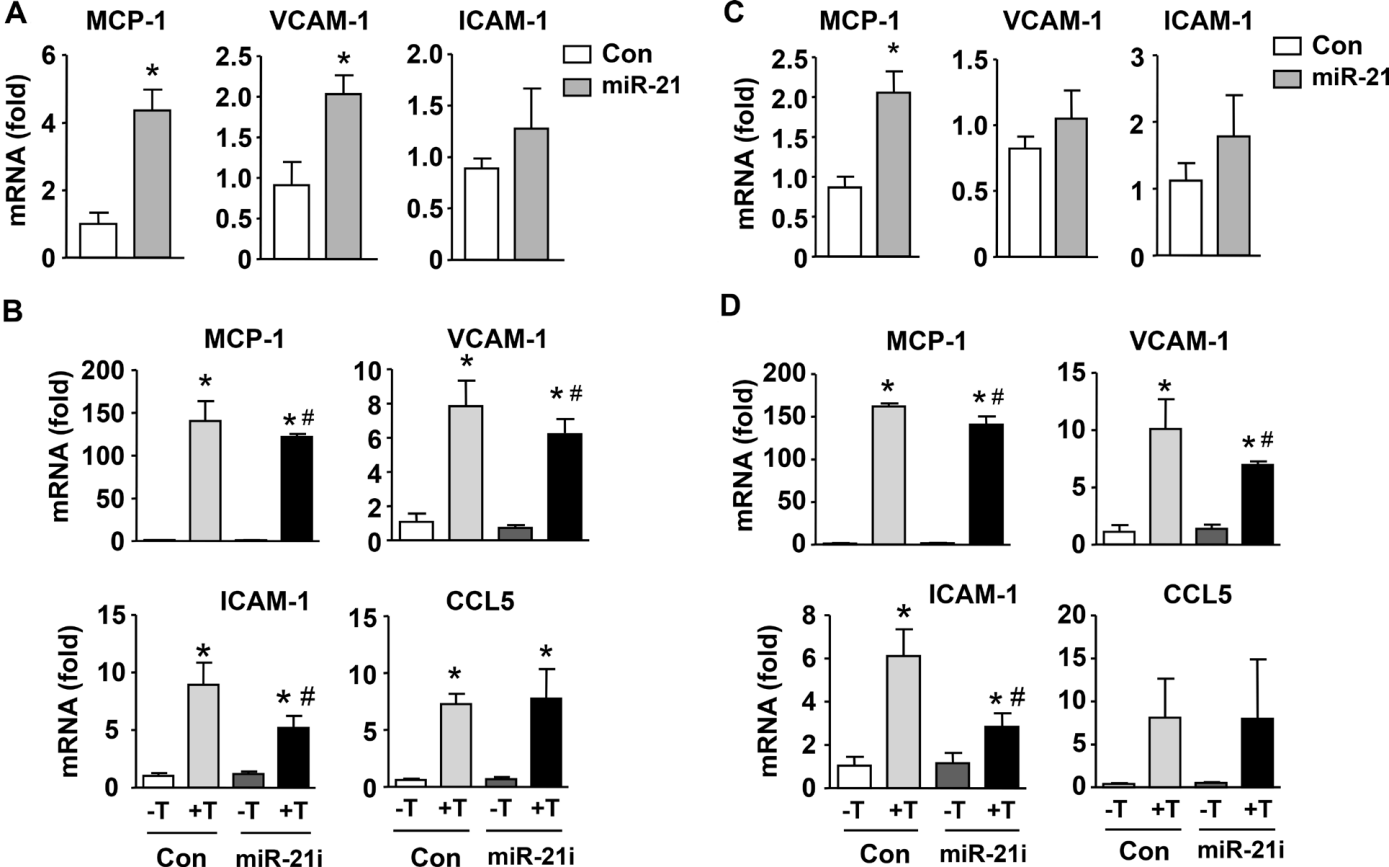
Role of miR-21 in mediating inflammatory responses in smooth muscle (**A** and **B**) and adventitial fibroblasts (**C** and **D**). (A and C) Effects of expression of a miR-21 mimic on mRNA levels of various inflammatory molecules as indicated as compared to scrambled control. (B and D) Effects of miR-21 antagomir (miR-21i) on expression levels of various inflammatory molecules in the absence and presence of TNF-a (T). Data are mean ± SD. *P < 0.05 versus Con, ^#^*P* < 0.05 versus Con+T, unpaired *t-test* or one-way ANOVA, *n* = 3.

Although a number of studies have shown that miR-21 contributes to the development of pathologic vascular remodeling, its precise roles in modulating inflammatory reactions in vascular cells remain to be defined. To address this question, we treated VSMCs with a miR-21 mimic, and measured the expressions of various inflammatory molecules. We showed that miR-21 mimic significantly enhanced the expressions of MCP-1 and VCAM-1, although the increase in ICAM-1 expression was not statistically significant (Figure 5A). Next we stimulated VSMCs with TNF-α, which significantly increased the expressions of MCP-1, VCAM-1 and ICAM-1; we demonstrated that pretreatment with miR-21 antagomir significantly attenuated TNF-induced inflammatory responses (Figure 5B). We also repeated these experiments in adventitial fibroblasts. We found that miR-21 mimic significantly increased the expression of MCP-1, but not VCAM-1 or ICAM-1 (Figure 5C). However, expressions of these inflammatory molecules stimulated by TNF-α were all attenuated by miR-21 antagomir (Figure 5D). In either VSMCs or fibroblasts, miR-21 antagomir showed no effects on the expression level of C-C motif ligand 5 (CCL5) (Figure 5B and 5D). These data together indicate that miR-21 overall has a pro-inflammatory role in vascular cells, particularly in the presence of TNF-α.

### Inhibiting miR-21 suppresses TNF-induced proliferation and migration in fibroblasts and VSMCs

**Figure 6:**
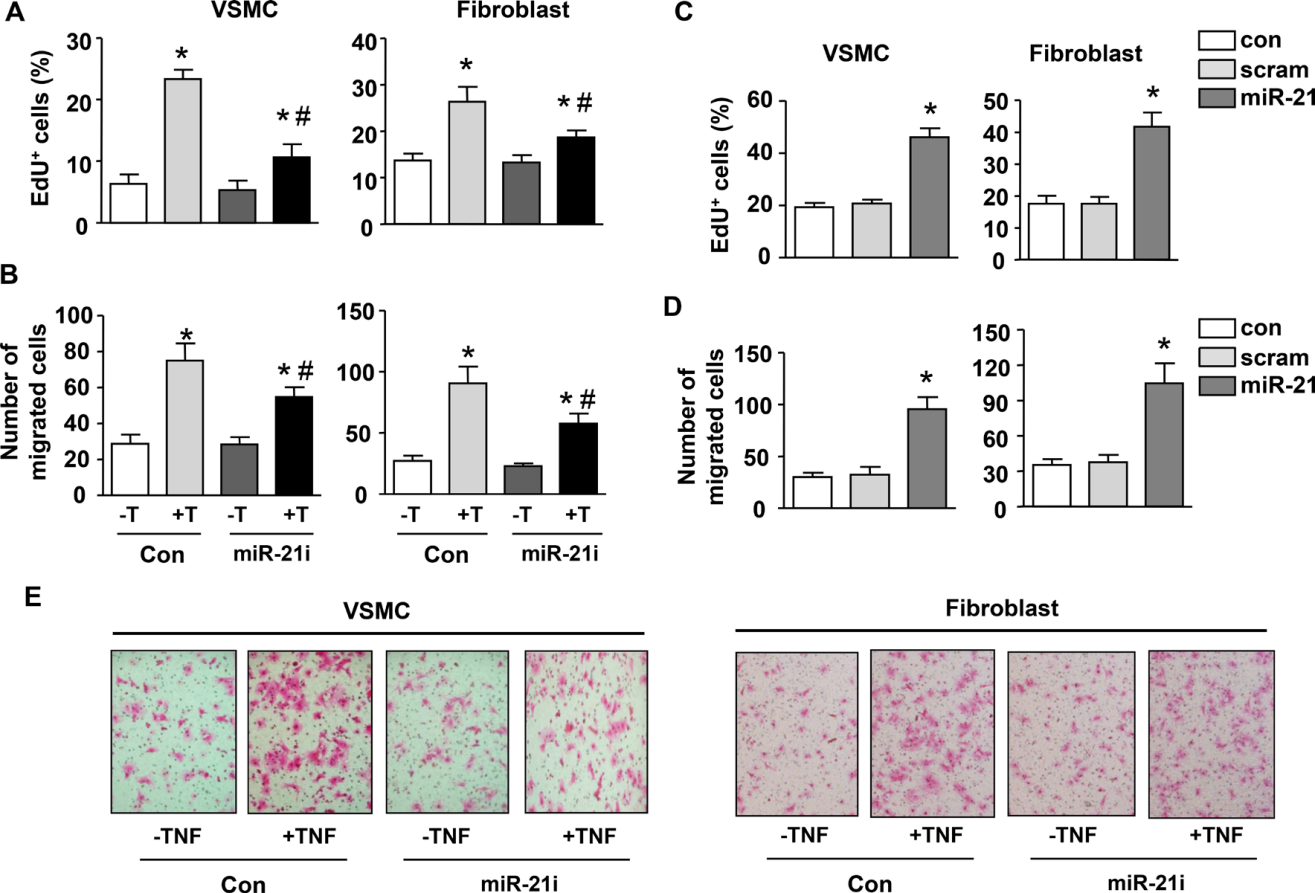
MiR-21 mediates TNF-induced proliferation and migration in vascular smooth muscle cells and fibroblasts (**A**) Effects of miR-21 antagomir (miR-21i) on serum-driven cell proliferation in the absence and presence of TNF-a (T). (**B**) Effects of miR-21i on cell migration activity in the absence and presence of TNF-a. (**C**) Serum-driven cell proliferation in untreated (Con), scrambled control (scram)-treated and miR-21 mimic-treated cells. (**D**) The migration activity of untreated (Con), scrambled control (scram)-treated and miR-21 mimic-treated cells. (**E**) Representative images showing the involvement of miR-21 in mediating TNF-stimulated migration in smooth muscle cells and fibroblasts. Cells were stained with eosin. Data are mean ± SD. *P < 0.05 versus Con, ^#^*P* < 0.05 versus Con+T, one-way ANOVA, *n* = 4.

We have established that TNF-α has a major role in mediating macrophage-stimulated miR-21 expression in vascular mural cells. To further confirm that miR-21 is a functional downstream effector of TNF-α in vascular cells, we measured cell proliferation and migration in response to TNF-α with and without miR-21 antagomir. We demonstrated that TNF-α enhanced proliferation and migration of both VSMCs and adventitial fibroblasts, which were attenuated by miR-21 antagomir (Figure 6A to 6B). The effects of miR-21 antagomir alone were minor. Conversely, we transfected VSMCs and fibroblasts with miR-21 mimic, and showed that miR-21 mimic significantly increased the proliferation and migration of both cells (Figure 6C to 6D). Representative images showing the role of miR-21 in mediating TNF-stimulated migration in VSMCs and fibroblasts were shown in Figure 6E.

## DISCUSSION

MiR-21 has been implicated in angioplasty-induced arterial neointima formation in various animal models [19, 27, 29, 30]. Using this established molecule as a test target, here we provided evidence suggesting that macrophages could promote transplantation-induced neointima formation by promoting miRNA expression in mural cells. This paracrine process may represent a novel alternative mechanism underlying the pathogenic actions of macrophages in arterial remodeling. Previously we found that macrophage accumulation in the tunica adventitia was an early pathological change preceding the development of neointima in transplanted rat aorta [14]. In the present study, we showed that macrophage depletion suppressed neointimal hyperplasia and reduced transplantation-induced vascular inflammation, supporting that macrophages had a pivotal role in the pathogenesis of transplant vasculopathy. This finding is consistent with an earlier report, in which the authors have demonstrated that macrophage depletion using carrageenan results in ~70% reduction in the development of neointima in transplanted murine hearts [32]. Nonetheless, the effects of carrageenan can be somehow non-specific. Hence, our data with clodronate liposomes further established the importance of macrophage infiltration in the pathogenesis of transplantation-induced neointima formation. Similar findings of adventitial macrophage infiltration prior to neointima formation are also reported in arteries after balloon angioplasty [33].

An interesting finding in the present study is that macrophage can stimulate miRNA expressions in vascular mural cells via a paracrine signaling mechanism. Specifically, using miR-21 as an example of vascular enriched miRNA [19, 27, 29, 30], we have identified that macrophages may promote miR-21 expression in VSMCs and adventitial fibroblasts via the liberation of TNF-α. We suggest that this process may represent a novel mechanism by which macrophages contribute to the development of pathological neointimal remodeling. Although it is well established that macrophage-derived cytokines can regulate vascular cell functions via their canonical signal transduction pathways, given the emerging evidence on the crucial roles of miRNAs in mediating vascular remodeling, we argue that the importance of macrophage-dependent paracrine upregulation of miRNA expression in vascular mural cells should not be under-recognized [34]. In addition, we have provided *in vivo* evidence showing that macrophage depletion reduces the expression level of miR-21, while blocking miR-21 functions mimics the effects of macrophage depletion on neointima formation and vascular inflammation, further supporting the above argument.

There is evidence that macrophages also express miR-21 [35, 36], and macrophage depletion reduces the abundance of macrophages in the aortic wall. To exclude the possibility that the reduction in miR-21 expression in the aorta after clodronate treatment was primarily a consequence of macrophage removal from the aortic wall, we independently measured miR-21 levels in the media and adventitia. In the present rat model, we confirmed that macrophages were not present in the medial layer throughout the experimental period. We further showed that miR-21 expression in the media was significantly reduced after clodronate treatment, suggesting that macrophage depletion could down-regulate miR-21 expression in SMCs. These data support that adventitial macrophages may enhance mural cell miR-21 expression via a paracrine mechanism. Given the pivotal role of proliferation and migration of medial SMCs in the development of transplant vasculopathy [3–5, 7], and the well documented detrimental effects of miR-21 in pathological vascular remodeling [19, 27, 29, 30, 37, 38], we suggest that the observed beneficial effects of macrophage depletion in aortic allografts are, at least in part, due to reduced miR-21 expression in vascular mural cells.

We have shown that miR-21 antagomir partially blocks the stimulating effects of TNF-a on these responses, supporting that miR-21 is a functional downstream effector of TNF-α in stimulating VSMC proliferation and migration. Moreover, our data confirm that miR-21 produces similar stimulating effects in adventitial fibroblasts, which may contribute to neointima formation in parallel with SMCs [39, 40]. Another novel finding in the present study is that miR-21, in addition to its actions on mural cell proliferation and migration, also exerts pro-inflammatory effects in vascular cells, especially in the presence of TNF-α. In this respect, previous studies in non-vascular cells have yielded varying results, suggesting that miR-21 has both pro-inflammatory and anti-inflammatory actions depending on the type of cells [41]. In contrast to its pro-inflammatory effects in vascular cells, most evidence indicates that miR-21 has an anti-inflammatory role in macrophages [35, 36]. Therefore, the vascular protective effects following miR-21 targeting reported in previous studies as well as in the present study are likely to be mediated mainly through its effects in vascular mural cells but not macrophages [19, 27, 29, 30, 38].

The current consensus is that macrophages may contribute to chronic rejection through the release of multiple inflammatory mediators, among which TNF-α is the most important one. Evidence is emerging suggesting that TNF-α may affect vascular cell functions by modulating the expression of different miRNA molecules [42, 43]. Consistent with our present results, Wei *et al*. have demonstrated that nuclear factor-kB, the direct downstream effector of TNF-α, can promote miR-21 expression in cardiomyocytes under oxidative stress [44]. These pieces of information together support the argument that miRNAs may act as important signaling modules in pathways of pro-inflammatory cytokines such as TNF-α [45], and these miRNAs may represent novel therapeutic targets for inhibiting tissue inflammation.

In summary, we have provided evidence suggesting that macrophages may promote transplantation-induced neointimal remodeling by stimulating miRNA expression in vascular mural cells, which in turn stimulates mural cell proliferation, migration and/or inflammation. Moreover, we have established that TNF-α has a major role in mediating this paracrine process. Nonetheless, since TNF-α may affect the expression of multiple miRNAs [46], a limitation of the present study is that we only selected miR-21 (a well documented pathogenic factor in vascular smooth muscle cells) as a target to prove the principle, whereas comparisons with other miRNAs were not carried out. Hence additional experiments are needed to further establish such a paracrine mechanism.

## MATERIALS AND METHODS

### Animal experiments

All animal study protocols were approved by the Institutional Animal Ethics Committee of Shandong University. Male F344 and Lewis inbred rats (weighing 220–270 g) were purchased from Charles River Laboratories (Beijing, China). Allograft aortic transplantation was performed as described previously using a cuff-based technique [14, 15]. Briefly, thoracic aortas from F344 rats were removed and inserted into the abdominal region of recipient Lewis rats between the levels of renal artery and iliac bifurcation. Neutral clodronate liposomes (from FormuMax Scientific, Palo Alto, CA, USA) were used for *in vivo* macrophage depletion. A dose of 1 mL of liposomes (250 mg/mL) per rat was given 3 days before the surgery via intra-peritoneal injection. Two supplementary doses of 1 mL each were given 24 hrs and 7 days after the surgery. Control animals were treated with the same dosage using control liposomes supplied by the manufacturer. For *in vivo* treatment with miR-21 antagomir, 50 μL of 20% (w/v) chilled F-127 pluronic gel (from Sigma-Aldrich, Shanghai, China) containing 20 μM of the antagomir (synthesized by GenePharma, Shanghai, China) or equivalent non-targeting control sequence was applied over the allograft aorta, and allowed to solidify at the body temperature.

### Morphometric analysis

Animals were sacrificed at various time points from day 1 to day 14 after transplantation. The vessel graft was removed and fixed in paraformaldehyde. Paraffin-embedded tissue sections were cut and stained with hematoxylin and eosin. Morphometric measurements of the average thickness of the intimal, medial and adventitial layers were carried out as described in our previous studies [14, 15].

### Immunohistochemistry

Immunohistochemistry was carried out as described previously [14, 47]. Briefly, sections were treated with 3% (v/v) hydrogen peroxide at 37°C for 10 min to quench the endogenous peroxidase activity. To retrieve antigens, the sections were heated in a citrate buffer (pH 6.0) in microwave oven for 15 min at high power. Sections were blocked with normal goat serum and incubated with the following primary antibodies: anti-CD3 (Abcam, Cambridge, UK), anti-CD68 (Boster, Wuhan, China), anti- proliferating cell nuclear antigen (PCNA) (Cell Signalling, Beverley, MA, USA), anti- monocyte chemoattractant protein-1 (MCP-1) (Abcam), anti- vascular cell adhesion molecule-1 (VCAM-1) and anti-intercellular adhesion molecule-1 (ICAM-1) (both from Proteintech, Wuhan, China).

### Culture of cell lines

NIH3T3 cells were cultured in Dulbecco's modified Eagle's medium (DMEM, from Corning, NY, USA) supplemented with 10% fetal bovine serum (FBS, Thermo Scientific, Waltham, MA, USA) and maintained in humidified atmosphere of 5% CO_2_ at 37^°^C. For cell treatments, recombinant transforming growth factor-β (TGF-β) (5 ng/mL), tumor necrosis factor-α (TNF-α) (5 ng/mL), vascular endothelial growth factor (VEGF)-165 (20 ng/mL), platelet-derived growth factor (PDGF) (10 ng/mL) or interleukin-6 (IL-6) (10 ng/mL) (all purchased from R&D Systems, Minneapolis, MN, USA) were added to the medium for 24 hrs. The macrophage cell line RAW264.7 was cultured in DMEM containing 10% FBS as described [48].

### Preparation of macrophage-conditioned medium

Tom prepare macrophage-conditioned medium, 2 × 10^5^ RAW264.7 cells were plated in 1 mL of culture medium in a 35-mm dish. Macrophages were treated with saline or 100 ng/mL of lipopolysaccharides (LPS, from Sigma) for 4 hrs, then the supernatant was discarded and cells washed with phosphate-buffered saline. Then cells were incubated in fresh DMEM for additional 24 hrs. The conditioned medium was collected by centrifugation and stored in aliquots at −80^°^C

### Isolation and culture of primary adventitial fibroblasts and vascular smooth muscle cells

Primary adventitial fibroblasts were isolated from aortic segments using the method described previously [14]. Cells were maintained in DMEM/F12 medium containing 10% FBS. VSMCs were isolated from rat aorta by enzymatic digestion and maintained in DMEM with 10% FBS as described [49].

### Enzyme-linked immunosorbent assay (ELISA)

The concentrations of TGF-β and TNF-α in the conditioned medium were determined using ELISA kits from Dakewe Biotech (Beijing, China) according to the manufacturer's protocol.

### Real-time quantitative polymerase chain reaction (qPCR)

To test how macrophage depletion affected miR-21 expression, we performed qPCR analysis using miRCURY LNA Universal cDNA Synthesis Kit and miR-21-specific primers (all from Exiqon, Vedbaek, Denmark) as described previously [50]. After tissue harvesting, the adventitial, medial and neointimal layers were dissected physically using microsurgical forceps, and homogenized in TRIzol reagent (Invitrogen, Carlsbad, CA). Because miR-21 was shown to be widely expressed in different cells, we performed PCR assays in medial and adventitial samples separately. U6 small nuclear RNA was used as the housekeeping gene.

For mRNA levels, reverse transcription was performed with PrimeScript RT reagents (TaKaRa, Kyoto, Japan) using 1 μg of total RNA; qPCR was performed using SYBR Green chemistry (SsoFas EvaGreen Supermix, from Bio-Rad, Hercules, CA, USA) as described [48]. The sequences of primers (forward and reverse respectively, all 3′ to 5′) were: GATCCCAATGAG TCGGCTG and TGGACCCATTCCTTATTGGGG for MCP-1; GGAAATGCCACCCTCACCT and TCAGAACA ACGGAATCCCCA for VCAM-1; GCCTGGGGTTGG AGACTAAC and CTGTCTTCCCCAATGTCGCT for ICAM-1. GAPDH was used as the housekeeping gene. The 2^−ΔΔCt^ method was used for data comparisons.

### Cell proliferation assays

Cell proliferation was determined by 5-ethynil-2’-deoxyuridine (EdU) assay using a kit from RiboBio (Guangzhou, China), according to the manufacturer's instructions.

### Cell migration assay

Cell migratory activities were assessed using Boyden chambers (BD Biosciences, San Jose, California, USA) as described previously [14, 51].

### Cell transfection

Transfection of primary fibroblasts and VSMCs was performed using X-tremeGENE siRNA Transfection Reagent (Roche, Shanghai, China). The final concentration of oligonucleotides was 50 nM. Constructs of miR-21 mimic and miR-21 antagomir, and corresponding scrambled controls, were purchased from Genepharma (Shanghai, China). All transfections were carried out in triplicate. Cells were assayed 48 hrs after transfection.

### Statistical analysis

Data were expressed as mean ± standard deviation (SD). Statistical analysis was performed using SPSS 17.0 software. Data were analyzed with unpaired *t-test* or one-way analysis of variance (ANOVA) followed by Tukey's test as appropriate (all two-tailed). *P* values less than 0.05 were considered to be statistically significant.

## SUPPLEMENTARY MATERIALS FIGURES AND TABLES



## Abbreviations

VSMC: vascular smooth muscle cell
miRNA: microRNA
PCNA: proliferating cell nuclear antigen
MCP-1: monocyte chemoattractant protein-1
VCAM-1: vascular cell adhesion molecule-1
ICAM-1: intercellular adhesion molecule-1
DMEM: Dulbecco's modified Eagle's medium
FBS: fetal bovine serum
TGF-β: transforming growth factor-β
TNF-α: tumor necrosis factor-α
VEGF: vascular endothelial growth factor
PDGF: platelet-derived growth factor
IL-6: interleukin-6
LPS: lipopolysaccharides
qPCR: quantitative polymerase chain reaction
ELISA: enzyme linked immunosorbent assay
ANOVA: analysis of variance
SD: standard deviation

## References

[1] Lindenfeld J, Miller GG, Shakar SF, Zolty R, Lowes BD, Wolfel EE, Mestroni L, Page RL, Kobashigawa J (2004). Drug therapy in the heart transplant recipient: part II: immunosuppressive drugs. Circulation.

[2] Wedel J, Bruneau S, Kochupurakkal N, Boneschansker L, Briscoe DM (2015). Chronic allograft rejection: a fresh look. Curr Opin Organ Transplant.

[3] Arora S, Gullestad L (2014). The challenge of allograft vasculopathy in cardiac transplantation. Curr Opin Organ Transplant.

[4] Mitchell RN (2004). Allograft arteriopathy: pathogenesis update. Cardiovasc Pathol.

[5] Mitchell RN, Libby P (2007). Vascular remodeling in transplant vasculopathy. Circ Res.

[6] Suzuki J, Isobe M, Morishita R, Nagai R (2010). Characteristics of chronic rejection in heart transplantation: important elements of pathogenesis and future treatments. Circ J.

[7] Pober JS, Jane-wit D, Qin L, Tellides G (2014). Interacting mechanisms in the pathogenesis of cardiac allograft vasculopathy. Arterioscler Thromb Vasc Biol.

[8] Hollis IB, Reed BN, Moranville MP (2015). Medication management of cardiac allograft vasculopathy after heart transplantation. Pharmacotherapy.

[9] Mennander A, Tiisala S, Halttunen J, Yilmaz S, Paavonen T, Hayry P (1991). Chronic rejection in rat aortic allografts. An experimental model for transplant arteriosclerosis. Arterioscler Thromb.

[10] Johnson P, Carpenter M, Hirsch G, Lee T (2002). Recipient cells form the intimal proliferative lesion in the rat aortic model of allograft arteriosclerosis. Am J Transplant.

[11] Li J, Liu S, Li W, Hu S, Xiong J, Shu X, Hu Q, Zheng Q, Song Z (2012). Vascular smooth muscle cell apoptosis promotes transplant arteriosclerosis through inducing the production of SDF-1alpha. Am J Transplant.

[12] Shears LL, Kawaharada N, Tzeng E, Billiar TR, Watkins SC, Kovesdi I, Lizonova A, Pham SM (1997). Inducible nitric oxide synthase suppresses the development of allograft arteriosclerosis. J Clin Invest.

[13] Thaunat O, Louedec L, Dai J, Bellier F, Groyer E, Delignat S, Gaston AT, Caligiuri G, Joly E, Plissonnier D, Michel JB, Nicoletti A (2006). Direct and indirect effects of alloantibodies link neointimal and medial remodeling in graft arteriosclerosis. Arterioscler Thromb Vasc Biol.

[14] Sun M, Ji J, Guo X, Liu W, Wang Y, Ma S, Hu W, Wang J, Jiang F (2016). Early adventitial activation characterized by NADPH oxidase expression and neovascularization in an aortic transplantation model. Exp Mol Pathol.

[15] Ji J, Xu F, Li L, Chen R, Wang J, Hu WC (2010). Activation of adventitial fibroblasts in the early stage of the aortic transplant vasculopathy in rat. Transplantation.

[16] Xu F, Ahmed AS, Kang X, Hu G, Liu F, Zhang W, Zhou J (2015). MicroRNA-15b/16 Attenuates Vascular Neointima Formation by Promoting the Contractile Phenotype of Vascular Smooth Muscle Through Targeting YAP. Arterioscler Thromb Vasc Biol.

[17] Liu YF, Spinelli A, Sun LY, Jiang M, Singer DV, Ginnan R, Saddouk FZ, Van Riper D, Singer HA (2016). MicroRNA-30 inhibits neointimal hyperplasia by targeting Ca(2+)/calmodulin-dependent protein kinase IIdelta (CaMKIIdelta). Sci Rep.

[18] Choe N, Kwon JS, Kim JR, Eom GH, Kim Y, Nam KI, Ahn Y, Kee HJ, Kook H (2013). The microRNA miR-132 targets Lrrfip1 to block vascular smooth muscle cell proliferation and neointimal hyperplasia. Atherosclerosis.

[19] Ji R, Cheng Y, Yue J, Yang J, Liu X, Chen H, Dean DB, Zhang C (2007). MicroRNA expression signature and antisense-mediated depletion reveal an essential role of MicroRNA in vascular neointimal lesion formation. Circ Res.

[20] Merlet E, Atassi F, Motiani RK, Mougenot N, Jacquet A, Nadaud S, Capiod T, Trebak M, Lompre AM, Marchand A (2013). miR-424/322 regulates vascular smooth muscle cell phenotype and neointimal formation in the rat. Cardiovasc Res.

[21] Yang Z, Zheng B, Zhang Y, He M, Zhang XH, Ma D, Zhang RN, Wu XL, Wen JK (2015). miR-155-dependent regulation of mammalian sterile 20-like kinase 2 (MST2) coordinates inflammation, oxidative stress and proliferation in vascular smooth muscle cells. Biochim Biophys Acta.

[22] Chen Q, Yang F, Guo M, Wen G, Zhang C, le A Luong, Zhu J, Xiao Q, Zhang L (2015). miRNA-34a reduces neointima formation through inhibiting smooth muscle cell proliferation and migration. J Mol Cell Cardiol.

[23] Cheng Y, Liu X, Yang J, Lin Y, Xu DZ, Lu Q, Deitch EA, Huo Y, Delphin ES, Zhang C (2009). MicroRNA-145, a novel smooth muscle cell phenotypic marker and modulator, controls vascular neointimal lesion formation. Circ Res.

[24] Liu X, Cheng Y, Zhang S, Lin Y, Yang J, Zhang C (2009). A necessary role of miR-221 and miR-222 in vascular smooth muscle cell proliferation and neointimal hyperplasia. Circ Res.

[25] Sheedy FJ, O’Neill LA (2008). Adding fuel to fire: microRNAs as a new class of mediators of inflammation. Ann Rheum Dis.

[26] Bravo-Egana V, Rosero S, Klein D, Jiang Z, Vargas N, Tsinoremas N, Doni M, Podetta M, Ricordi C, Molano RD, Pileggi A, Pastori RL (2012). Inflammation-Mediated Regulation of MicroRNA Expression in Transplanted Pancreatic Islets. J Transplant.

[27] Wang F, Zhao XQ, Liu JN, Wang ZH, Wang XL, Hou XY, Liu R, Gao F, Zhang MX, Zhang Y, Bu PL (2012). Antagonist of microRNA-21 improves balloon injury-induced rat iliac artery remodeling by regulating proliferation and apoptosis of adventitial fibroblasts and myofibroblasts. J Cell Biochem.

[28] McDonald RA, White KM, Wu J, Cooley BC, Robertson KE, Halliday CA, McClure JD, Francis S, Lu R, Kennedy S, George SJ, Wan S, van Rooij E (2013). miRNA-21 is dysregulated in response to vein grafting in multiple models and genetic ablation in mice attenuates neointima formation. Eur Heart J.

[29] Wang D, Deuse T, Stubbendorff M, Chernogubova E, Erben RG, Eken SM, Jin H, Li Y, Busch A, Heeger CH, Behnisch B, Reichenspurner H, Robbins RC (2015). Local MicroRNA Modulation Using a Novel Anti-miR-21-Eluting Stent Effectively Prevents Experimental In-Stent Restenosis. Arterioscler Thromb Vasc Biol.

[30] McDonald RA, Halliday CA, Miller AM, Diver LA, Dakin RS, Montgomery J, McBride MW, Kennedy S, McClure JD, Robertson KE, Douglas G, Channon KM, Oldroyd KG (2015). Reducing In-Stent Restenosis: Therapeutic Manipulation of miRNA in Vascular Remodeling and Inflammation. J Am Coll Cardiol.

[31] Wei Y, Nazari-Jahantigh M, Chan L, Zhu M, Heyll K, Corbalan-Campos J, Hartmann P, Thiemann A, Weber C, Schober A (2013). The microRNA-342-5p fosters inflammatory macrophage activation through an Akt1- and microRNA-155-dependent pathway during atherosclerosis. Circulation.

[32] Kitchens WH, Chase CM, Uehara S, Cornell LD, Colvin RB, Russell PS, Madsen JC (2007). Macrophage depletion suppresses cardiac allograft vasculopathy in mice. Am J Transplant.

[33] Hanke H, Hassenstein S, Ulmer A, Kamenz J, Oberhoff M, Haase KK, Baumbach A, Gown AM, Karsch KR (1994). Accumulation of macrophages in the arterial vessel wall following experimental balloon angioplasty. Eur Heart J.

[34] Wei Y, Schober A, Weber C (2013). Pathogenic arterial remodeling: the good and bad of microRNAs. Am J Physiol Heart Circ Physiol.

[35] Caescu CI, Guo X, Tesfa L, Bhagat TD, Verma A, Zheng D, Stanley ER (2015). Colony stimulating factor-1 receptor signaling networks inhibit mouse macrophage inflammatory responses by induction of microRNA-21. Blood.

[36] Barnett RE, Conklin DJ, Ryan L, Keskey RC, Ramjee V, Sepulveda EA, Srivastava S, Bhatnagar A, Cheadle WG (2016). Anti-inflammatory effects of miR-21 in the macrophage response to peritonitis. J Leukoc Biol.

[37] Stein JJ, Iwuchukwu C, Maier KG, Gahtan V (2014). Thrombospondin-1-induced vascular smooth muscle cell migration and proliferation are functionally dependent on microRNA-21. Surgery.

[38] Sarkar J, Gou D, Turaka P, Viktorova E, Ramchandran R, Raj JU (2010). MicroRNA-21 plays a role in hypoxia-mediated pulmonary artery smooth muscle cell proliferation and migration. Am J Physiol Lung Cell Mol Physiol.

[39] Siow RC, Mallawaarachchi CM, Weissberg PL (2003). Migration of adventitial myofibroblasts following vascular balloon injury: insights from *in vivo* gene transfer to rat carotid arteries. Cardiovasc Res.

[40] Li G, Chen SJ, Oparil S, Chen YF, Thompson JA (2000). Direct *in vivo* evidence demonstrating neointimal migration of adventitial fibroblasts after balloon injury of rat carotid arteries. Circulation.

[41] Sheedy FJ (2015). Turning 21: Induction of miR-21 as a Key Switch in the Inflammatory Response. Front Immunol.

[42] Li S, Chen H, Ren J, Geng Q, Song J, Lee C, Cao C, Zhang J, Xu N (2014). MicroRNA-223 inhibits tissue factor expression in vascular endothelial cells. Atherosclerosis.

[43] Ruan W, Xu JM, Li SB, Yuan LQ, Dai RP (2011). Effects of down-regulation of microRNA-23a on TNF-alpha-induced endothelial cell apoptosis through caspase-dependent pathways. Cardiovasc Res.

[44] Wei C, Li L, Kim IK, Sun P, Gupta S (2014). NF-kappaB mediated miR-21 regulation in cardiomyocytes apoptosis under oxidative stress. Free Radic Res.

[45] McCoy CE (2011). The role of miRNAs in cytokine signaling. Front Biosci (Landmark Ed).

[46] Meyer SU, Thirion C, Polesskaya A, Bauersachs S, Kaiser S, Krause S, Pfaffl MW (2015). TNF-alpha and IGF1 modify the microRNA signature in skeletal muscle cell differentiation. Cell Commun Signal.

[47] Wang JL, Ma SQ, Li L, Liu GQ, Hu WC, Ma R (2013). Correlation of inflammatory cells in adventitia and formation and extending of atherosclerotic lesions in coronary artery of apolipoprotein E gene knockout mice. Chin J Physiol.

[48] Liu FF, Wu X, Zhang Y, Wang Y, Jiang F (2014). TRAIL/DR5 signaling promotes macrophage foam cell formation by modulating scavenger receptor expression. PLoS One.

[49] Jiang F, Guo N, Dusting GJ (2009). 3′,4′-Dihydroxyflavonol down-regulates monocyte chemoattractant protein-1 in smooth muscle: role of focal adhesion kinase and PDGF receptor signalling. Br J Pharmacol.

[50] Liu D, Han L, Wu X, Yang X, Zhang Q, Jiang F (2014). Genome-wide microRNA changes in human intracranial aneurysms. BMC Neurol.

[51] Wang Y, Yan F, Ye Q, Wu X, Jiang F (2016). PTP1B inhibitor promotes endothelial cell motility by activating the DOCK180/Rac1 pathway. Sci Rep.

