# Supplementary material for: Macrophage-stimulated microRNA expression in mural cells promotes transplantation-induced neointima formation
Source: Oncotarget. 2017 Mar 16;8(18):30100–11. doi: 10.18632/oncotarget.16279 (PMC5444729; doi:10.18632/oncotarget.16279)
Supplement: Supplementary file 1 [file oncotarget-08-30100-s001.pdf]

Macrophage-stimulated microRNA expression in mural cells promotes transplantation-induced neointima formation

Supplementary Materials

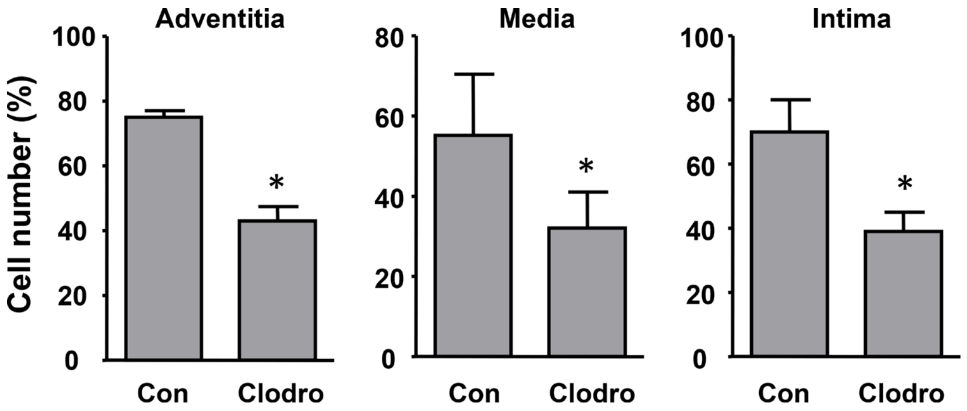

Supplementary Figure 1: Quantitative data showing that clodronate treatment reduced the abundance of PCNA$^+$ cells in all three layers of the vessel wall. Data were mean ± SD. **P* < 0.05 versus control , unpaired *t*-test, *n* = 6 per group.

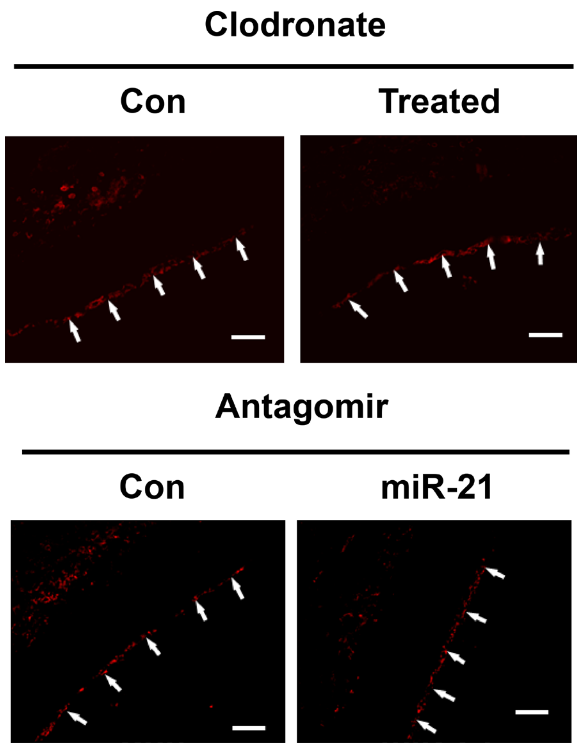

Supplementary Figure 2: Fluorescent staining of endothelium using DyLight 594-labeled *Lycopersicon* lectin in untreated and treated aortas, showing that clodronate liposome or miR-21 antagomir had no obvious effect on the integrity of the endothelial layer (arrows). Bar = 50 µm.

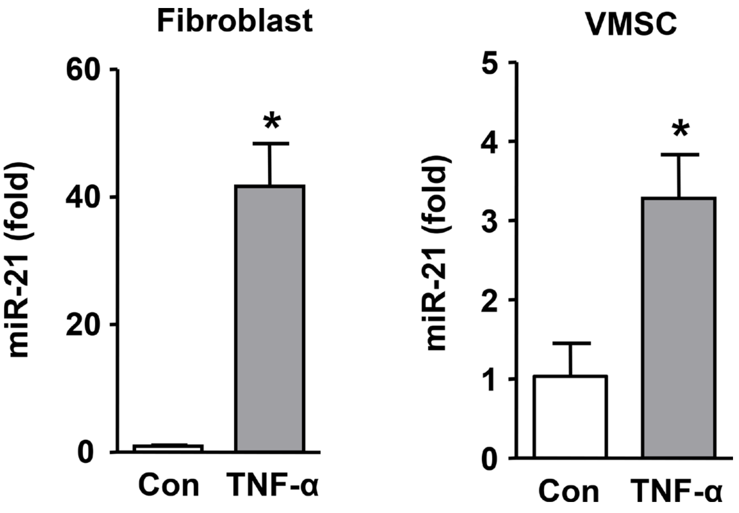

Supplementary Figure 3: PCR results showing that TNF-α treatment upregulated miR-21 expressions in primary fibroblasts and VSMCs. *$P < 0.05$ *vs* control, unpaired *t*-test, $n = 3$.
